# Encapsulation of Few-Layer MoS_2_ in the Pores of Mesoporous Carbon Hollow Spheres for Lithium-Sulfur Batteries

**DOI:** 10.3390/nano9091247

**Published:** 2019-09-03

**Authors:** Yunyan Zhao, Qianyu Zhuang, Wenda Li, Hongrui Peng, Guicun Li, Zhonghua Zhang

**Affiliations:** College of Materials Science and Engineering, Qingdao University of Science and Technology, Qingdao 266042, China

**Keywords:** ultrathin few-layer MoS_2_ nanosheets, mesoporous carbon hollow sphere, multi-fold structural, lithium sulfur batteries

## Abstract

Integrating a highly conductive carbon host and polar inorganic compounds has been widely reported to improve the electrochemical performances for promising low-cost lithium sulfur batteries. Herein, a MoS_2_/mesoporous carbon hollow sphere (MoS_2_/MCHS) structure has been proposed as an efficient sulfur cathode via a simple wet impregnation method and gas phase vulcanization method. Multi-fold structural merits have been demonstrated for the MoS_2_/MCHS structures. On one hand, the mesoporous carbon hollow sphere (MCHS) matrix, with abundant pore structures and high specific surface areas, could load a large amount of sulfur, improve the electronical conductivity of sulfur electrodes, and suppress the volume changes during the repeated sulfur conversion processes. On the other hand, ultrathin multi-layer MoS_2_ nanosheets are revealed to be uniformly distributed in the mesoporous carbon hollow spheres, enhancing the physical adsorption and chemical entrapment functionalities towards the soluble polysulfide species. Having benefited from these structural advantages, the sulfur-impregnated MoS_2_/MCHS cathode presents remarkably improved electrochemical performances in terms of lower voltage polarization, higher reversible capacity (1094.3 mAh g^−1^), higher rate capability (590.2 mAh g^−1^ at 2 C), and better cycling stability (556 mAh g^−1^ after 400 cycles at 2 C) compared to the sulfur-impregnated MCHS cathode. This work offers a novel delicate design strategy for functional materials to achieve high performance lithium sulfur batteries.

## 1. Introduction

The development of lithium ion (Li-ion) battery technologies cannot meet the ever-growing demand of electric vehicles and automobiles due to their relatively low energy density (387 Wh kg^−1^ for LiCoO_2_/C) and their relatively high cost. Fortunately, lithium–sulfur (Li-S) batteries have an ultrahigh theoretical energy density (2600 Wh kg^−1^) and high theoretical specific capacity of 1675 mA h g^−1^ based on the multielectron conversion electrochemistry between lithium and elemental sulfur [1,2]. In addition, elemental sulfur, the main cathode material of Li–S batteries, is eco-friendly and low-cost [3]. Considering those factors, Li-S batteries are considered as next-generation high-energy batteries [4,5,6]. However, several intrinsic problems from sulfur cathodes are impeding the commercial application of lithium-sulfur battery technologies. Firstly, the intrinsic electronic insulating nature of the charged-state elemental S_8_ and discharge product (Li_2_S_2_ and Li_2_S) leads to slow kinetics, low rate capability, and serious electrochemical polarizations. Secondly, dramatic volume expansion (about 80%) during the conversion reaction from S to Li_2_S brings about pulverization of the electrodes and short electrode service lifetime [7,8]. Last but not least, the intermediated products of lithium polysulfides (Li_2_S_n_, 4 ≤ n ≤ 8) can migrate to the lithium metal anode as its solubility in the organic liquid electrolyte, leading to serious loss of active material, low coulombic efficiency, and poor cycling stability [9,10,11].

To address the aforementioned issues, significant efforts have been made in designing cathodes, such as preparing all kinds of carbon–sulfur composites, conductive polymers-sulfur composites, metal organic framework (MOF)–sulfur composites, and so on [12,13,14,15,16,17,18,19]. Conductive carbon has been one of the most appropriate hosts for sulfur to date [20]. Nanostructured carbon materials may load a large amount of sulfur and relieve the volume expansion of sulfur on account of their large specific surface area, great pore volume, and controllable pore size distribution. However, the “shuttle effect” of polysulfides cannot be solved in carbon-sulfur material due to poor affinity between nonpolar carbon and polar polysulfides [21,22,23]. Recently, some polar materials, such as metal oxides, transition metal disulfides, polymer materials, and polar heteroatoms, have been paid much attention because of the strong chemical interactions with soluble lithium polysulfides [24,25,26,27,28,29,30]. Especially, polar molybdenum disulfide (MoS_2_) has advantages of high safety, low cost, and being a simple synthetic method [31], Nevertheless, the poor conductivity of these polar materials hinders the polysulfide conversion, resulting in electrochemical performance degradation. Hence, the combined use of conductive carbon materials and polar materials is usually accepted in establishing sulfur electrode hosts [32,33,34]. However, the electron transport pathway still can be blocked sometimes because of the phase segregation caused by the lack of surface affinity between carbon materials and polar materials [35]. Therefore, it is very meaningful to design sulfur electrode hosts with hassle-free cladding for polar nanometer materials.

**Scheme 1:**
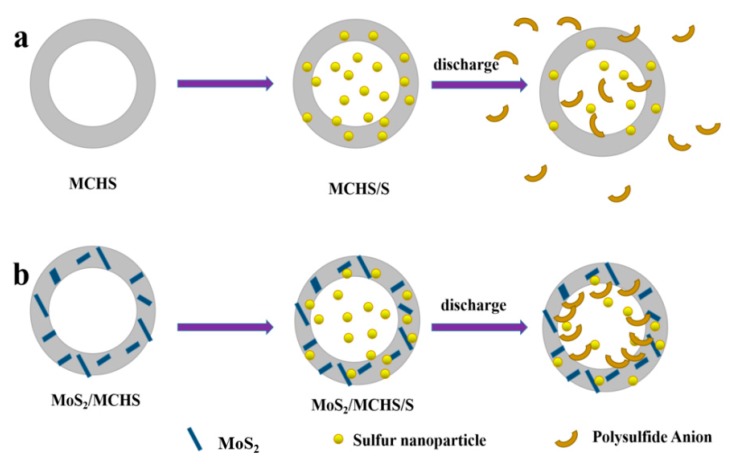
Proposed adsorption mechanism of polysulfides for (**a**) the MCHS/S and (**b**) MoS_2_/MCHS/S electrodes.

In this study, a delicately-designed hierarchical MoS_2_/mesoporous carbon hollow sphere (MoS_2_/MCHS) structure is developed by a simple wet impregnation method and gas phase vulcanization method as a sulfur host material for Li-S batteries. The encapsulation of MoS_2_ in the pores of mesoporous carbon hollow spheres (MCHS) possesses multi-fold structural merits of both the chemical entrapment of MoS_2_ and high conductivity of MCHS. Specifically, on the path that polysulfides must cross, special MoS_2_ defense lines are built. Polar MoS_2_ can chemically trap polysulfides via strong chemical bonds, so that polysulfides are largely fixed and remained inside the conductive MCHS (Scheme 1). Additionally, the conductive network provides electrons for redox reaction in a timely manner to facilitate polysulfide conversion kinetics. The unique structure not only provides adequate storage space to sulfur, but also increases sulfur utilization. Therefore, the MoS_2_/MCHS/S electrode shows improved cycling stability and better rate capability in comparison to the MCHS/S electrode. The as-proposed unique MoS_2_/MCHS host structure provides a new method for designing high performance functional electrode materials for energy application.

## 2. Materials and Methods

### 2.1. Preparation of MoS_2_/MCHS Composite

All of the chemicals were used as received. Mesoporous carbon hollow spheres used in this work were prepared by the surfactant-free method using tetrapropyl orthosilicate, resorcinol, and formaldehyde [36]. The MoS_2_/MCHS composite was synthesized using a simple wet impregnation method and gas phase vulcanization method. Typically, phosphomolybdic acid hydrate (34 mg) was dissolved in 5 mL of ethanol under vigorous stirring for 30 min. Then, 0.1 g of MCHS powder treated with nitric acid was dispersed in the above aqueous solution by ultrasonic method for 30 min, and dried under vacuum at 60 °C for 12 h. Finally, the sample and sulfur powder in a 1:2 mass ratio were heated under Ar atmosphere at 600 °C for 2 h with a heating rate of 5 °C/min to obtain MoS_2_/MCHS composite.

### 2.2. Preparation of the MoS_2_/MCHS/S and MCHS/S Composites

The MoS_2_/MCHS/S and MCHS/S composites were synthesized through a classic melt-diffusion strategy. Briefly, sulfur powder and MoS_2_/MCHS (or MCHS) in a 3:1 mass ratio were mixed uniformly in a mortar. The mixture was heated at 155 °C for 12 h in a 50 mL Teflon-lined stainless-steel autoclave. After that, the mixture was heated at 200 °C for 1 h with a heating rate of 10 °C/min in a quartz tube filled with N_2_ to distribute the sulfur uniformly in MoS_2_/MCHS composite (or MCHS).

### 2.3. Material Characterizations

The morphology of the samples was analyzed by performing field-emission scanning electron microscopy (SEM; JSM-6700F, JEOL Ltd., Tokyo, Japan), transmission electron microscopy (TEM; JEM-2100F, JEOL Ltd., Tokyo, Japan), and high-resolution transmission electron microscopy (HRTEM; Tecnai G20, FEI corp., Hillsboro, OR, USA). Thermogravimetric analysis (TGA) was performed on a TG-DTA 6200 LAB SYS analyzer (SII NanoTechnology Inc., Tokyo, Japan) under N_2_ and air atmosphere. The pore-size distribution was obtained by nitrogen adsorption–desorption measurement on a micromeritics ASAP 2020 analyzer (Micromeritics Instrument corp., Atlanta, GA, USA) at 77 K. The composition of the samples was determined by X-ray diffraction (XRD; D-max-gA, Rigaku corp., Tokyo, Japan) with Cu K_α_ radiation from 5° to 80° (2θ). X-ray photoelectron spectroscopy (XPS) was performed using a PHI 550 spectrometer (Perkin-Elmer corp., Waltham, MA, USA) with Al K_α_ radiation.

### 2.4. Electrochemical Measurements

The electrochemical tests were conducted on standard CR2032-type coin cells assembled in an argon filled glove box. The cathode was fabricated by mixing 70 wt.% MoS_2_/MCHS/S (or MCHS/S), with 20 wt.% carbon black (Super-P) and 10 wt.% poly(vinyl difluoride) (PVDF) in 1-methyl-2-pyrrolidinone (NMP), and the mixture was cast onto a pure Al foil. The mass loading of active materials on Al foil was about 0.5 mg cm^−2^. The electrochemical performances of electrodes with a high mass loading of about 1.6 mg cm^−2^ were also investigated. Besides, a lithium metal foil was served as the counter electrode and separated by a Celgard 2500 membrane separator. The electrolyte was a solution of 1.0 M bis(trifluoromethane)sulfonimide lithium salt and 0.1 M lithium nitrate in dimethyl ether (DME)/dioxolane (DOL) (volume ratio: 1:1). The galvanostatic discharge-charge experiments were carried out using a CT2001A battery tester (Land Electronic Co., Ltd., Wuhan, China) over a voltage range of 1.5 V–3.0 V (vs. Li/Li^+^). Cyclic voltammetry (CV) tests were conducted on an Autolab PGSTAT302N electrochemical workstation at a scan rate of 0.1 mV s^−1^.

## 3. Results and Discussion

### 3.1. Structure and Morphology

**Figure 1:**
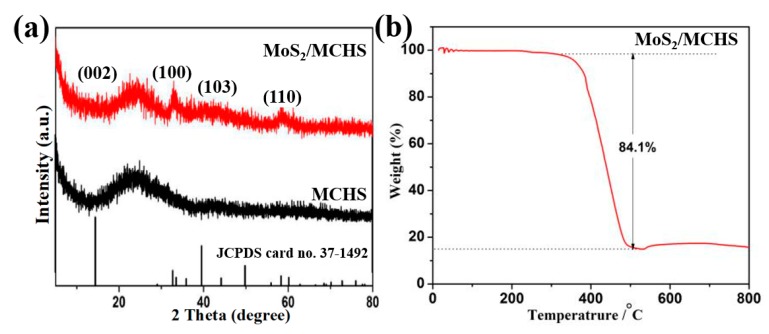
(**a**) X-ray diffraction patterns of the MoS_2_/MCHS and MCHS structures. (**b**) Heat curve of MoS_2_/MCHS composite structure.

The crystalline structure of both the MoS_2_/MCHS and MCHS structures was evaluated with XRD (Figure 1a). The broad diffraction at around 2θ = 20–30° corresponds to amorphous carbon characteristic peaks of MCHS. Three typical diffraction peaks at 33°, 39°, and 58° can be attributed to the (100), (103), and (110) plans of the MoS_2_ phase (JCPDS 37–1492), respectively [37]. An invisible peak of the (002) plane (2θ = 14°) suggests the presence of “graphene-like” layers that are less than five layers. This result agrees with the results of HRTEM [38]. To determine the content of MoS_2_ in the MoS_2_/MCHS structures, a TGA test was performed from room temperature to 800 °C in air (Figure 1b). The MoO_3_ residue after TGA comes from the oxidation of MoS_2_, so the proportion of MoS_2_ in the MoS_2_/MCHS structures was approximately 17.5%.

**Figure 2:**
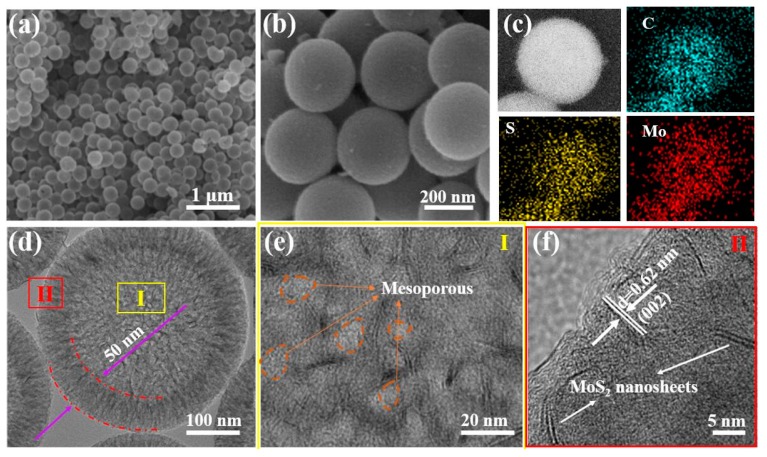
(**a**,**b**) Scanning electron microscopy image of MoS_2_/MCHS. (**c**) Energy dispersive spectrometer mapping images of the MoS_2_/MCHS composite structures. (**d**) Transmission electron microscopy images and (**e**,**f**) High-resolution transmission electron microscopy of MoS_2_/MCHS.

The morphologies of the MoS_2_/MCHS and MCHS structures were observed with SEM and TEM images. The MCHS structures were prepared by one-pot, surfactant-free polymerization method, which exhibits a rough-surfaced spherical structure with an average diameter of 320 nm (Figure S1). As shown in Figure 2a, the shape and size of the MoS_2_/MCHS structures are similar to those of the MCHS structures. Compared with the MCHS structures, the surface roughness of the MoS_2_/MCHS structures decreases, which may be caused by the reduced pore diameter under the sulfidation process. Besides, there is no obvious layered structure of MoS_2_ on the surface of the MCHS structures. To clarify the distribution of MoS_2_ in carbon shell, EDS element mapping is exhibited in Figure 2c. As can be seen, the C, Mo, and S elements are evenly distributed in the sphere. TEM images in Figure 2d and Figure S2a,b reveal that both the MoS_2_/MCHS and MCHS structures have similar radial porous shells, the thickness of which is about 50 nm. The radial pore channels and hollow morphology of both the MoS_2_/MCHS and MCHS structures are convenient for sulfur impregnation. Additionally, such a structure can also buffer volume changes of sulfur and shorten the ion or electron diffusion distance during the charge-discharge process. The HRTEM images were investigated to prove the existence of MoS_2_. Unlike the HRTEM images of MCHS (Figure S2c,d), there are some ultrathin MoS_2_ multi-layer crystals uniformly distributed in the MoS_2_/MCHS structures (Figure 2e,f). Figure 2f reveals the shell of MoS_2_/MCHS possesses abundant mesopores. Figure 2f indicates that the interplanar spacing of crystallites is 0.62 nm, which corresponds to the lattice spacing of MoS_2_ (002) plane [39]. Layered MoS_2_ is successfully encapsulated in the pores of the MCHS structures, evidenced in the HRTEM images. Additionally, layered MoS_2_ completely surrounded by conductive carbon may facilitate polysulfide conversion kinetics, and thus increase sulfur utilization.

**Figure 3:**
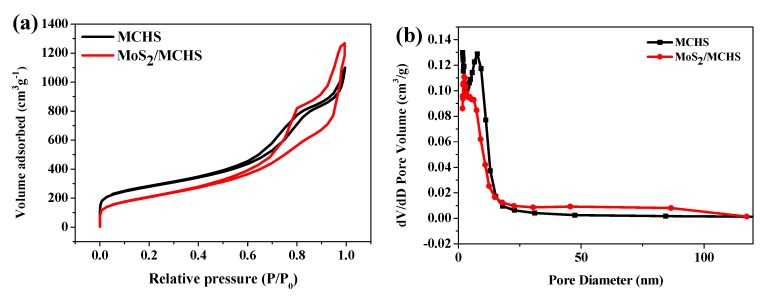
Nitrogen adsorption–desorption isotherm curves (**a**) and corresponding pore size distribution plots (**b**) of the MoS_2_/MCHS and MCHS structures.

To investigate the pore structures after introducing MoS_2_, nitrogen adsorption-desorption isotherms measurements were conducted. Figure 3a presents two similar N_2_ adsorption-desorption isotherms, which show mixed features of type-II isotherm and type-IV isotherm. Type-IV isotherm is characterized by the presence of significant hysteresis loops. Type-II isotherm represents the macropores material, while Type-IV isotherm represents the mesoporous structure. The pore size distribution plots were obtained by the Barrett-Joyner-Halenda (BJH) method (Figure 3b). The surface area and pore volume of the materials are listed in Table S1. After the introduction of MoS_2_, the main peak of the distribution plots shifts 6 nm towards a smaller pore-size (2 nm), indicating that the encapsulation of MoS_2_ in the pores of the MCHS structures leads to reduced pore diameter. In addition, the surface area of the MoS_2_/MCHS structure (727.32 m^2^ g^−1^) is slightly smaller than that of the MCHS structure (941.62 m^2^ g^−1^), suggesting a slight change of specific surface area produced by the addition of MoS_2_. Fortunately, the MoS_2_/MCHS structures basically maintain a large specific surface area and a high pore volume, which is beneficial to the infiltration of elemental sulfur and the transport of lithium ions. The pore structures before and after sulfur impregnation are compared in Figure S3 to determine the distribution of sulfur. The pore volume becomes smaller at any pore diameter after sulfur impregnation, due to the S distribution across the entire sphere.

**Figure 4:**
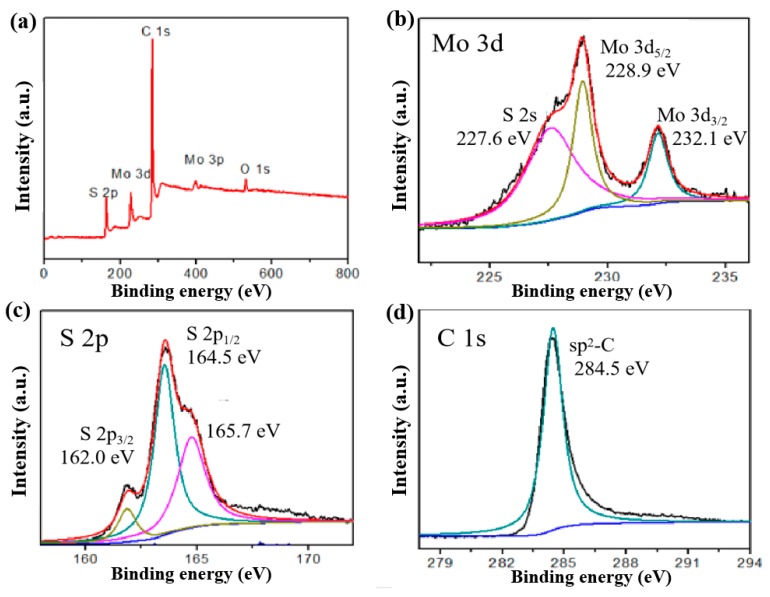
X-ray photoelectron spectroscopy spectrum of the MoS_2_/MCHS structures (**a**). High-resolution XPS spectra of Mo 3d-S 2s (**b**), S 2p (**c**), and C 1s (**d**).of the MoS_2_/MCHS structures.

The chemical composition of the MoS_2_/MCHS structures was investigated by XPS measurements. The XPS spectrum in Figure 4a clearly indicates that the MoS_2_/MCHS structures are composed of the Mo, S, C, and O elements, suggesting the existence of oxygen function groups on the surface of the material. The binding energies of Mo 3d_5/2_ and Mo 3d_3/2_ are located at 228.9 and 232.1 eV, demonstrating the presence of Mo^4+^ (Figure 4b) [40]. Additionally, the peak at 227.6 eV could be attributed to the S 2s orbital of MoS_2_ [41]. In the high-resolution spectrum of S 2p, the peaks at 162 and 164.5 eV are assigned to S 2p_3/2_ and S 2p_1/2_, which are associated with the existence of divalent sulfide ions (S^2−^). The 165.7 eV peaks may result from the presence of the bridging disulfide S_2_^2−^. As shown in Figure 4d, the peaks at 284.5 eV are due to the C = C bond in the MCHS structures. The results indicate that the MoS_2_/MCHS structures were successfully prepared in this research.

Figure S4a,b show that the morphology of both the MCHS and MoS_2_/MCHS structures were not obviously changed after sulfur impregnation. TGA data were also used to analyze the weight content and thermal stability of sulfur in the MoS_2_/MCHS/S composites, which were obtained between room temperature and 400 °C in N_2_ atmosphere (Figure S5). Considering that the sublimation of elemental sulfur is the reason for the weight loss, the contents of sulfur in both the MCHS/S and MoS_2_/MCHS/S composites are 71.5 and 72.6 wt %, respectively. In addition, the better thermal stability and sulfur retention ability of the MoS_2_/MCHS/S composites are observed due to the higher initial decomposition temperature and lower decomposition rate in early decay. The presence of MoS_2_ not only does not decrease the storage space of sulfur, but can load more sulfur on account of the stronger absorption.

A simple visual adsorption test was conducted to verify the interactive activity of the MoS_2_/MCHS structures and the soluble lithium polysulfide (Figure S6). The Li_2_S_6_ solution (0.01 M) with reddish brown color in Figure S6c is prepared by dissolving a stoichiometric amount of sulfur and Li_2_S in a 1:1 (*v*/*v*) DOL/DME solvent. The solution color changes from reddish brown to yellow after the addition of the MCHS structures owing to the physical absorptivity towards lithium polysulfides (Figure S6b). In sharp contrast, the solution is almost completely discolored after adding the MoS_2_/MCHS structures to the lithium polysulfide solution (Figure S6a). This might be responsible for the synergistic effects from the physical adsorption of the mesoporous carbon hollow spheres and the chemical bond between polar MoS_2_ and polysulfide. The strong interaction between the MoS_2_/MCHS structures and lithium polysulfide is beneficial for suppressing the “shuttle effect” of polysulfide and the destruction of Li anodes.

### 3.2. Electrochemical Performance

**Figure 5:**
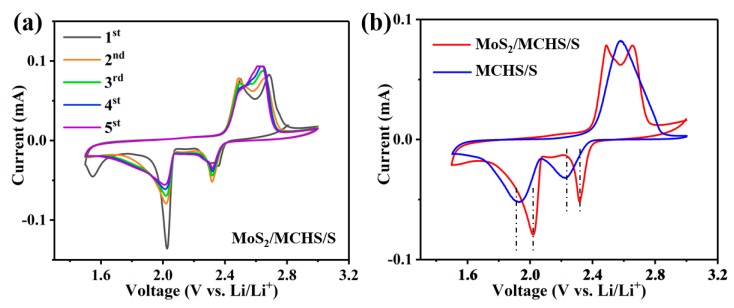
Typical Cyclic voltammetry (CV) curves of (**a**) the MoS_2_/MCHS/S cathode and (**b**) CV curves of the MoS_2_/MCHS/S and MCHS/S electrodes at a scan rate of 0.1 mV s^−1^ in the second cycle.

The typical CV curves of both the MoS_2_/MCHS/S cathodes and MCHS/S cathodes were measured over a voltage range of 1.5–3.0 V at a scan rate of 0.1 mV s^−1^ (Figure 5a and Figure S7). In Figure 5a, the CV curves of the MoS_2_/MCHS/S cathode show two representative reduction peaks at 2.03 and 2.32 V, corresponding to the step-wise reduction of S_8_ to the long-chain polysulfides (Li_2_S_n_, 4 ≤ n ≤ 8) and short-chain polysulfides (Li_2_S_2_/Li_2_S), respectively [41]. The reverse distinguishable oxidation peaks located at 2.49 and 2.68 V are associated with the formation of long-chain polysulfides and elemental sulfur, respectively, suggesting good reaction kinetics. In contrast, two merging oxidation peaks at around 2.52 V are observed in the CV curves of the MCHS/S cathode (Figure S7), indicating the two-step delithiation process. Furthermore, higher peak current and larger CV enclosed area of the MoS_2_/MCHS/S cathode also indicate the faster electrochemical kinetics (Figure 5b). The potential gap between oxidation and reduction peaks of the MoS_2_/MCHS/S cathodes (0.46 V) are lower than that of the MCHS/S cathodes (0.73 V), which illustrates a lower cell polarization and faster Li ions diffusion in the MoS_2_/MCHS/S cathode [42]. The above analyses suggest that the uniform dispersion of MoS_2_ in the MCHS matrix facilitates the capture of polysulfides and boosts the conversion rates of polysulfides.

**Figure 6:**
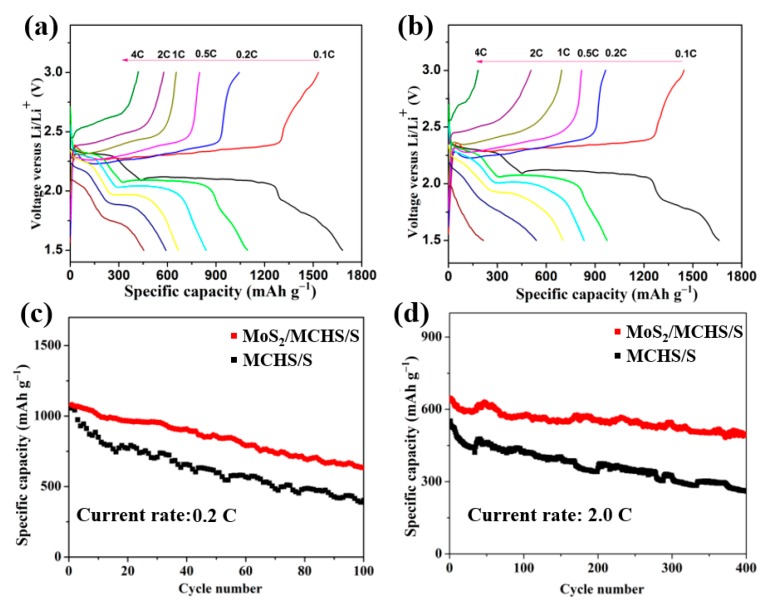
The galvanostatic charge and discharge curves of the MoS_2_/MCHS/S electrode (**a**) and MCHS/S electrode (**b**) at various currents. Cycling performance of the MCHS/S and MoS_2_/MCHS/S cathodes at 0.2 C (**c**) and 2 C (**d**).

The galvanostatic charge–discharge profiles of both the MoS_2_/MCHS/S electrode and the MCHS/S electrode at various currents are shown in Figure 6a,b. Two representative discharge plateaus can be observed in the curves of both the MoS_2_/MCHS/S electrode and MCHS/S electrode. At the current rate of 0.1 C, the upper discharge flat at 2.3 V is related to the transformation from sulfur to long-chain polysulfides, while the lower plateau at 2.1 V versus Li/Li^+^ is attributed to the formation of short-chain lithium polysulfides [43]. This result is in agreement with the CV analysis. In Figure 6b, the low potential of around 1.8 V corresponds to the conversion from Li_2_S_2_ to Li_2_S [44]. With the increase of the current rate, the voltage of the discharge plateau is more negative and the voltage of the charge flat is more positive, indicating greater polarization. It is noticeable that the polarization of the MoS_2_/MCHS/S cathode is smaller than that of the MCHS/S cathode, especially at high current rate. The clearly visible voltage platform of the MoS_2_/MCHS/S composite can be observed even at the 4 C rate, suggesting a fast electrode reaction. Meanwhile, in comparison to the MCHS/S cathode, the MoS_2_/MCHS/S cathode has a longer plateau due to the strong interaction between soluble polysulfides and MoS_2_, as well as high utilization percentage of sulfur.

The rate capabilities of both the MoS_2_/MCHS/S cathode and the MCHS/S cathode are displayed in Figure S8. The discharge capacities of the MoS_2_/MCHS/S cathodes are 1680.6, 1094.3, 838.6, 667.6, 590.2, and 453.9 mAh g^−1^ at rates of 0.1, 0.2, 0.5, 1, 2, and 4 C, respectively. When the current rate returns to 0.1 C, the specific capacity of the MoS_2_/MCHS/S cathode is maintained at a high reversible capacity of 1037.3 mAh g^−1^, implying a good reversibility. Compared with the MCHS/S electrode, the MoS_2_/MCHS/S electrode remarkably displays an enhanced specific capacity at high current rates (two times higher at 4 C).

Cycling performances of the MCHS/S and MoS_2_/MCHS/S cathodes at the 0.2 C rate are compared in Figure 6c. The MCHS/S cathode exhibits a lower initial specific capacity (1056.2 mAh g^−1^) and a severe capacity fading rate of 59.86% after 100 cycles. One possible reason is that the “shuttle effect” caused by soluble polysulfide occurs in this cathode system. In contrast, the MoS_2_/MCHS/S cathode shows a higher discharge of 1079.5 mAh g^−1^ and a reduced capacity decay rate of 37.40%, indicating that MoS_2_ can indeed trap the dissolved polysulfide and effectively suppress the “shuttle effect”. The electrochemical performance of electrodes with a high mass loading of about 1.6 mg cm^−2^ was also investigated in Figure S9. The MoS_2_/MCHS/S cathodes with a relatively high mass loading exhibited comparable specific capacity and cycle performance. Figure S9 compares the cycling performances of the MCHS/S and MoS_2_/MCHS/S cathodes with a high mass loading at the 1.0 C rate. The MCHS/S cathode shows a capacity fading rate of 27.08% after 100 cycles, while the MoS_2_/MCHS/S cathode displays an obviously improved capacity fading rate of 5.07% after 100 cycles. The MoS_2_/MCHS/S cathodes exhibit less capacity decay than the MoS_2_/MCHS/S cathodes at both high mass loading and low mass loading conditions, indicating the superiority of cathode design strategy. It is worth noting that the MoS_2_/MCHS/S electrode displays a significantly improved long-term cycling stability at a higher current rate (2 C). As shown in Figure 6d, the MoS_2_/MCHS/S cathode delivers a high initial discharge capacity of 639.9 mAh g^−1^ and a high capacity retention rate of 77.65% is obtained after 400 cycles. However, for the MCHS/S cathode, its initial specific capacity and capacity retention rate are 551.8 mAh g^−1^ and 47.3%, respectively. Compared with the MCHS/S cathode, the electrochemical performances of the MoS_2_/MCHS/S cathode have been unambiguously improved regarding the aspects of rate capability and cycling performance. These results can be ascribed to the unique structure of the MoS_2_/MCHS/S composite. Specifically, by introducing polar MoS_2_ in the pores of the carbon shell, the polysulfides, which are generated during the charge and discharge process, can be trapped due to the chemical interaction. Meanwhile, the conductive carbon is tight around the ultrathin multi-layer MoS_2_ nanosheet and can provide enough electrons for the redox reaction to facilitate polysulfide conversion. It is worth mentioning that there are enough diffusion paths of Li ions in the MoS_2_/MCHS/S composites, considering the abundant pore structures. Additionally, as Figure S10 shows, the MoS_2_/MCHS/S spheres can retain their morphology after cycling, demonstrating that our material can suppress the volume changes during the repeated sulfur conversion processes. Hence, the delicately-designed MoS_2_/MCHS/S structure is beneficial to accelerate the sulfur conversion kinetics and the synergistic effect could be achieved between physical adsorption of the MCHS structure and the chemical trap of polar MoS_2_, both of which contribute to increased sulfur utilization, improved rate capability, and superior cycling performance.

## 4. Conclusions

In conclusion, a simple wet impregnation method and gas phase vulcanization method have been developed to synthesize the unique MoS_2_/MCHS structure as a sulfur host for lithium–sulfur batteries. The intriguing aspect of the architecture is the encapsulation of ultrathin, multi-layer MoS_2_ nanosheets in the pores of mesoporous carbon hollow spheres. On one hand, conductive mesoporous carbon hollow spheres can improve the electrical conductivity of sulfur cathodes, shorten the Li ion diffusion distance, and buffer volume changes of sulfur. On the other hand, polar MoS_2_ nanosheets uniformly distributed in the mesoporous carbon shell act as the polysulfide-trapping center, where soluble polysulfides are largely anchored and transformed. As expected, the MoS_2_/MCHS/S electrode exhibits a high specific capacity (1094.3 mAh g^−1^, 0.2 C), good rate capability (590.2 mAh g^−1^, 2 C), and excellent cycling stability (77.65 % capacity retention after 400 cycles at 2 C). The unique electrode design strategy and innovative structure are expected to provide new ideas and versatile methods for fabrication of layered transition metal compounds, which may provide more opportunities for the development of advanced sulfur-based and metal sulfide-based electrode materials.

## Supplementary Materials

The following are available online at https://www.mdpi.com/2079-4991/9/9/1247/s1, Figure S1: SEM images of the MCHS, Figure S2: TEM images of MCHS (a,b); HTEM images of MCHS (c,d), Figure S3: Pore size distribution plots of the MoS2/MCHS and MoS2/MCHS/S structures, Figure S4: SEM images of (a) the MCHS/S and (b) MoS2/MCHS/S, Figure S5: Heat curve of MCHS/S and MoS2/MCHS/S composite electrode, Figure S6: Visual adsorption test for MoS2/MCHS (a) and MCHS (b). Digital photograph of Li2S6 solutions (c), Figure S7: CV curves of the MCHS/S cathode at scan rate of 0.1 mV s^−1^, Figure S8: Rate performances at various cycling rates of the MCHS/S and MoS2/MCHS/S composite electrode, Figure S9: SEM images of the MoS2/MCHS/S electrode at 1C after 10 cycles, Figure S10: SEM images of the MoS2/MCHS/S electrode at 1C after 10 cycles, Table S1: The surface area and pore volume of different materials.
