# Supplementary material for: Encapsulation of Few-Layer MoS2 in the Pores of Mesoporous Carbon Hollow Spheres for Lithium-Sulfur Batteries
Source: Nanomaterials (Basel). 2019 Sep 3;9(9):1247. doi: 10.3390/nano9091247 (PMC6780156; doi:10.3390/nano9091247)
Supplement: Supplementary file 1 [file nanomaterials-09-01247-s001.pdf]

nanomaterials

MDPI

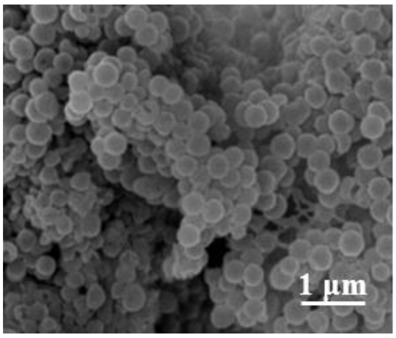

Figure 1. SEM images of the MCHS.

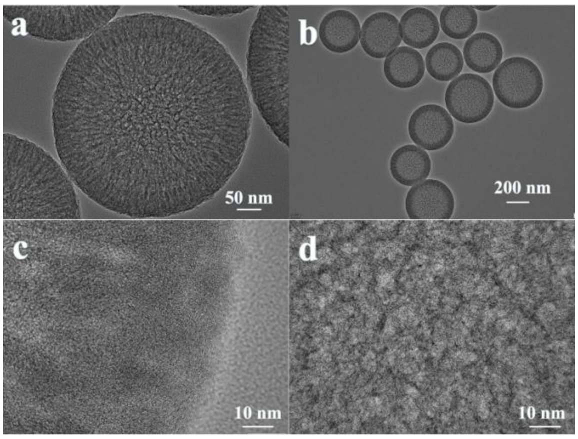

Figure 2. TEM images of MCHS (a,b); HTEM images of MCHS (c,d).

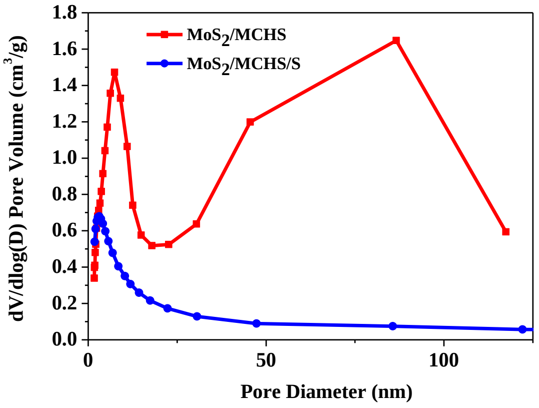

Figure 3. Pore size distribution plots of the MoS_2/MCHS and MoS_2/MCHS/S structures.

Table 1. surface area and pore volume of different materials.

sample	surface area ($m^2\,g^{-1}$)	pore volume (cm^3g^{-1})
MCHS	941.62	1.73
MoS_2/MCHS	727.32	1.96
MoS_2/MCHS/S	396.50	0.63

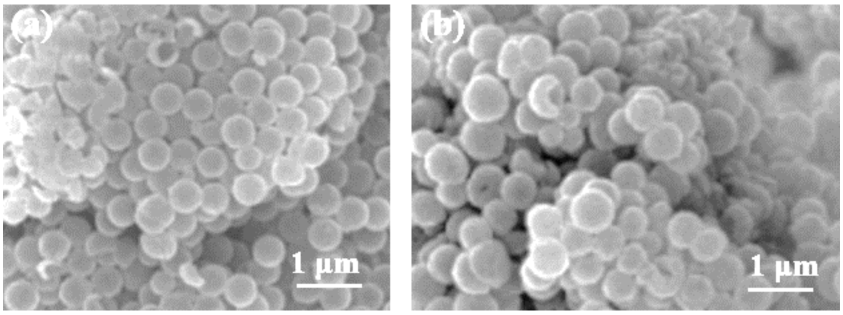

Figure 4. SEM images of (a) the MCHS/S and (b) MoS_2/MCHS/S.

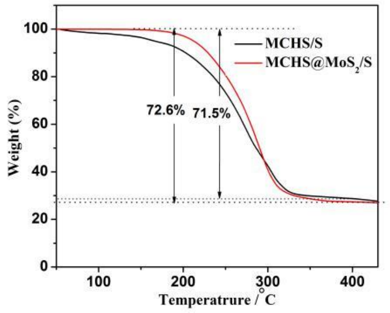

Figure 5. Heat curve of MCHS/S and MoS_2/MCHS/S composite electrode.

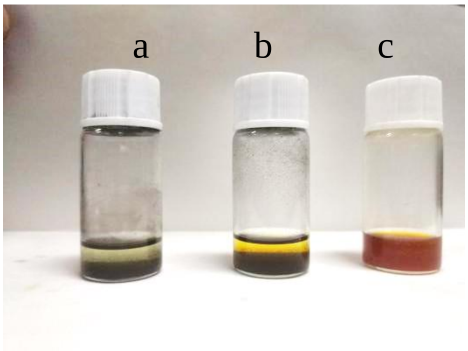

Figure 6. Visual adsorption test for MoS_2/MCHS (a) and MCHS (b). Digital photograph of Li_2S_6 solutions (c).

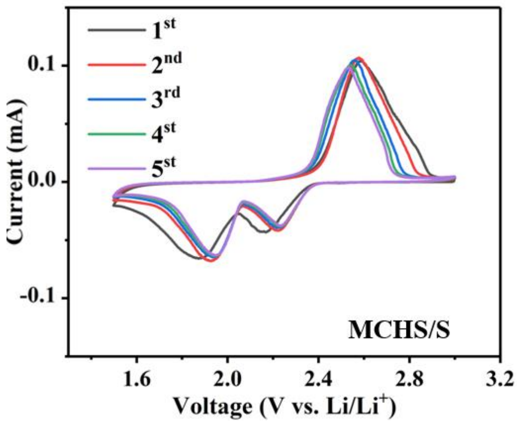

Figure 7. CV curves of the MCHS/S cathode at scan rate of 0.1 mV s^{-1}.

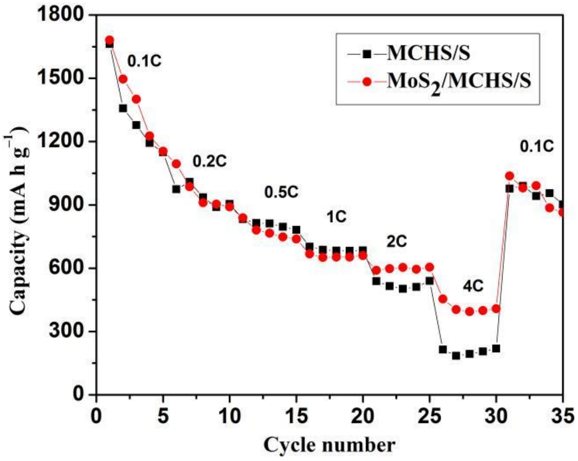

Figure 8. Rate performances at various cycling rates of.the MCHS/S and MoS_2/MCHS/S composite electrode.

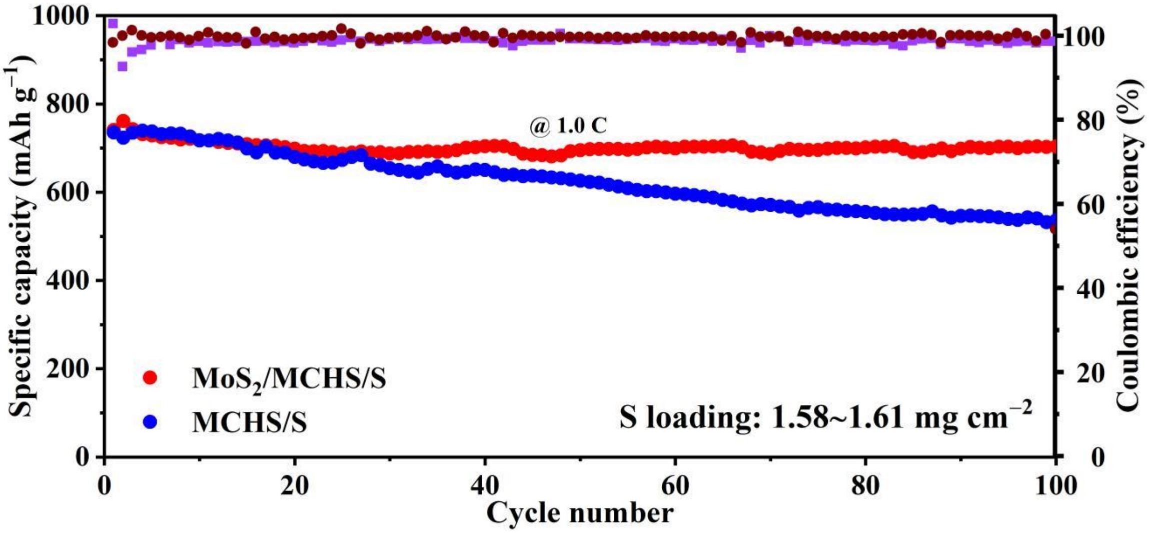

Figure 9. Cycling performance of the MCHS/S and MoS_2/MCHS/S cathodes (a high mass loading of about 1.6mg cm^{-2}) at 1 C .

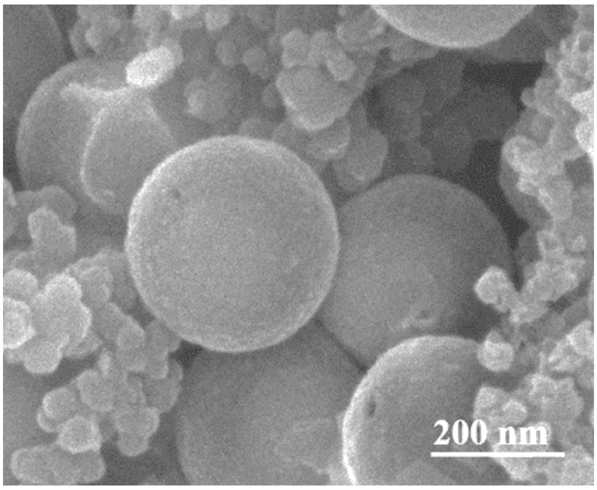

Figure 10. SEM images of the MoS_2/MCHS/S electrode at 1C after 10 cycles.

The rate capability of MoS_2/MCHS/S composite electrode and MCHS/S composite electrode were also evaluated by cycling at various current densities from 0.1 to 4 C (Figure S8). The first cycle capacity of the MCHS/S composite electrode and MoS_2/MCHS/S composite electrode at C-rates of 0.1 C, 0.2 C, 0.5 C, 1 C, 2 C and 4 C were shows in figure 6. The MCHS composite electrode exhibits initial specific discharge capacity values for different C-rates were 1661.9, 973.9, 832.6, 702.4, 539.1, 214.5 mAh g^{-1}, while the MoS_2/MCHS/S composite electrode exhibits high initial specific discharge capacity values for different C-rates were 1680.6, 1094.3, 838.6, 667.6, 590.2, and 453.9 mAh g^{-1}, and when the discharge rate reply from 4 C to 0.1 C, the specific capacity can quickly reply to high specific capacity, reaching 1037.3 mAh g^{-1}. It shows that the MoS_2/MCHS/S composite electrode has good cycling performance. According to the test results, the rate capability of MCHS composite electrode was fades rapidly, and the specific capacity decay was more pronounced at high current densities, due to an apparent dissolution of sulfide during charge–discharge process. By sharp contrast, the MoS_2/MCHS/S composite electrode has a better rate performance at the rates of 2C and 4C. This is due to the composite structure of MoS_2 layers built in the inner of the mesoporous carbon hollow spheres that suppression the "shuttle effect" of sulfur and obtain a high sulfur utilization. There is an interaction between the polysulfide ions generated during the charge and discharge process of the lithium-sulfur battery with MoS_2 layers, which provides the effect of chemical interaction of sulfur species, and its composite structure doesn't affect the conductivity of the carbon sphere surface. The mesoporous carbon hollow spheres provide a storage space for sulfur and the synergistic effect of the physical entrapment, while three-dimensional and porous structures are conducive to the promotion of Li^+ and electron transport.
